# Current Perspectives and Progress in Preoperative Portal Vein Embolization with Stem Cell Augmentation (PVESA)

**DOI:** 10.1007/s12015-024-10719-1

**Published:** 2024-04-13

**Authors:** Allan John R. Barcena, Tyler C. Owens, Sophie Melancon, Isias Workeneh, Hop S. Tran Cao, Jean-Nicolas Vauthey, Steven Y. Huang

**Affiliations:** 1 Department of Interventional Radiology, Division of Diagnostic Imaging, The University of Texas MD Anderson Cancer Center, 1400 Pressler St, Unit, Houston, TX 1471, 77030 United States; 2 College of Medicine, University of the Philippines Manila, Manila, NCR 1000 Philippines; 3 Department of Surgical Oncology, Division of Surgery, The University of Texas MD Anderson Cancer Center, Houston, TX 77030 United States

**Keywords:** Future liver remnant, Hematopoietic stem cells, Liver hypertrophy, Mesenchymal stem Cells, Portal vein embolization, Portal vein ligation, PVESA

## Abstract

Portal vein embolization with stem cell augmentation (PVESA) is an emerging approach for enhancing the growth of the liver segment that will remain after surgery (i.e., future liver remnant, FLR) in patients with liver cancer. Conventional portal vein embolization (PVE) aims to induce preoperative FLR growth, but it has a risk of failure in patients with underlying liver dysfunction and comorbid illnesses. PVESA combines PVE with stem cell therapy to potentially improve FLR size and function more effectively and efficiently. Various types of stem cells can help improve liver growth by secreting paracrine signals for hepatocyte growth or by transforming into hepatocytes. Mesenchymal stem cells (MSCs), unrestricted somatic stem cells, and small hepatocyte-like progenitor cells have been used to augment liver growth in preclinical animal models, while clinical studies have demonstrated the benefit of CD133 + bone marrow–derived MSCs and hematopoietic stem cells. These investigations have shown that PVESA is generally safe and enhances liver growth after PVE. However, optimizing the selection, collection, and application of stem cells remains crucial to maximize benefits and minimize risks. Additionally, advanced stem cell technologies, such as priming, genetic modification, and extracellular vesicle-based therapy, that could further enhance efficacy outcomes should be evaluated. Despite its potential, PVESA requires more investigations, particularly mechanistic studies that involve orthotopic animal models of liver cancer with concomitant liver injury as well as larger human trials.

## Background

Liver cancers represent the third leading cause of cancer death worldwide, and their aggressive nature and late detection often result in a poor prognosis [1]. For many individuals with primary and secondary liver cancers, liver resection offers a potential cure. However, many patients who would otherwise undergo curative liver resection do not have enough remaining functional liver to safely carry out major resection. This situation places patients at a high risk of life-threatening hepatic insufficiency due to minimal postoperative functional liver volume.

Portal vein ligation (PVL) and portal vein embolization (PVE) are methods developed in the mid-1980s to increase the future liver remnant (FLR) volume prior to resection by obstructing portal blood flow to the liver segments slated for removal [2]. PVL is typically performed by a surgeon either during open surgery or laparoscopy, while PVE is performed percutaneously by an interventional radiologist. Our group favors PVE over PVL because it tends to induce less inflammation in the porta hepatis, potentially enhancing the safety of subsequent major hepatectomies. Makuuchi et al. first described PVE in 1984, building on early 20th-century animal studies that demonstrated the growth of non-ligated liver segments following portal vein branch ligation [2]. Notably, about 70% of patients undergoing PVE can proceed to resection within six weeks of the procedure [3, 4].

**Fig. 1:**
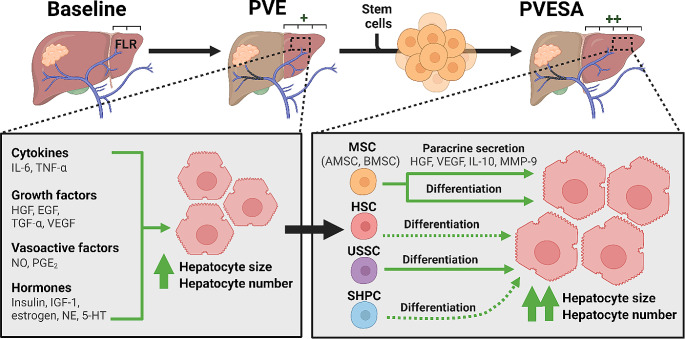
Schematic diagram of factors and mechanisms involved in PVE and PVESA. The growth of hepatocytes following PVE is mediated by several cytokines, growth factors, vasoactive substances, and hormones. On the other hand, PVESA enhances the FLR growth achieved with PVE through additional paracrine effects and differentiation. The differentiation of HSCs and SHPCs to hepatocytes has been suggested, but it requires further investigation. *Abbreviations*: 5-HT, serotonin; EGF, epidermal growth factor; FLR, future liver remnant; HGF, hepatocyte growth factor; HSC, hematopoietic stem cell; IGF-1, insulin-like growth factor 1; IL, interleukin; MMP-9, matrix metalloproteinase 9; MSC, mesenchymal stem cell; NE, norepinephrine; NO, nitric oxide; PGE_2_, prostaglandin E_2_; PVE, portal vein embolization; PVESA, portal vein embolization with stem cell augmentation; SHPC, small hepatocyte-like progenitor cell; TGF-α, transforming growth factor alpha; TNF-α, tumor necrosis factor alpha; USSC, unrestricted somatic stem cell; VEGF, vascular endothelial growth factor. The figure has been created using Biorender.com

Despite the use of portal vein occlusion strategies, post-hepatectomy liver failure remains the leading cause of morbidity and mortality after resection [3, 5]. To further augment FLR growth following PVL or PVE, adjunctive procedures, such as associated liver partition and PVL for staged hepatectomy (ALPPS) and liver venous deprivation (LVD), have been developed [6]. ALPPS is a two-pronged approach that combines complete transection of the liver along the falciform ligament and right PVL. However, it has been associated with significantly higher morbidity and mortality [7]. On the other hand, LVD, which combines PVE and hepatic venous embolization, has shown comparable intra-operative and post-operative complication rates with standalone PVE [8]. While these procedures may provide improvements in FLR growth, the outcomes for many patients with liver cancer remain severely limited by underlying chronic liver injury. In this clinical scenario, adjunctive intrahepatic administration of stem cells, in combination with PVE is a promising solution. In this review, we discuss the challenges of increasing the volume and quality of the FLR using PVE as a standalone procedure and the potential of PVE with stem cell augmentation (PVESA) to enhance the benefits seen with PVE. Various stem cell types have been utilized in preclinical and clinical research (Fig. 1), and the most recent data and outlook for each are also discussed.

## Current Clinical Use of Preoperative PVE or PVL

PVE is currently the standard method for increasing FLR volume prior to liver resection [5]. By directing portal blood flow away from the part of the liver that will be resected, FLR growth may be induced. PVE is generally associated with minimal procedure-related mortality and is well-tolerated by patients [9, 10]. Unfortunately, up to 30% of patients who receive PVE will experience insufficient liver growth to allow for major liver resection [3, 4]. The degree of liver growth achieved with PVE depends on the degree of liver disease present in the patient, as well as the technical aspects of the PVE procedure.

### Clinical Utility of PVE

For patients with liver malignancy, surgical resection or liver transplantation remain the best treatment options. However, liver transplantation is unavailable to many because of a scarcity of liver donors, while the presence of chronic liver damage in a large percentage of patients with liver cancer precludes surgical resection. FLR volume is an important determinant of resectability as it can predict post-hepatectomy liver failure and may constitute the difference between curative treatment and malignant progression. Maximizing the capacity to enhance FLR volume directly results in an increase in the number of lives saved from liver malignancy by expanding the cohort of patients who can undergo safe liver resection. For patients with normal healthy liver parenchyma, an FLR volume standardized to the patient’s body surface area of ≥ 20% is required. A higher FLR volume (30–40%) is recommended if chronic liver injury is present (e.g., chemotherapy-associated liver damage, liver fibrosis, or cirrhosis) [3, 11, 12]. For those with insufficient FLR volume or limited functional capacity because of concurrent liver dysfunction, PVE is the gold standard for inducing an increase in FLR. Hence, PVE allows some patients with liver malignancies to undergo curative resection. The most common malignancies for which PVE is indicated prior to resection are hepatocellular carcinoma (HCC), cholangiocarcinoma (CCA), and metastatic colorectal cancer (mCRC). For patients with intrahepatic colorectal metastasis, resection can offer significantly prolonged survival and a superior prognosis with regard to further disease progression [13, 14].

### Mechanism of Liver Growth from PVE

The liver has long been known to have significant regenerative capacity. The first published instance of liver regeneration following PVE was a 1920 paper describing the compensatory growth of the non-embolized sections of the liver in rabbits following occlusion of the other portal veins [15]. The mechanisms of hepatic growth after PVE appear to be similar to those seen in partial hepatectomy (PH). However, the more sudden change in hemodynamics observed in PH contributes to more rapid activation of liver proliferation compared to PVE. The rapid increase in hepatic portal venous blood flow into the non-embolized lobe increases shear and circumferential stress on sinusoidal endothelial cells, hepatocytes, and Kupffer cells, initiating a cascade of signaling events that leads to an increase in pro-proliferative cytokines (e.g., interleukin-6 and tumor necrosis factor alpha), growth factors (e.g., hepatocyte growth factor [HGF], epidermal growth factor [EGF], transforming growth factor alpha, and vascular endothelial growth factor [VEGF]), vasoactive factors (e.g., nitric oxide [NO] and prostaglandin E_2_ [PGE_2_]), and hormones (e.g., insulin, insulin-like growth factor 1, estrogen, norepinephrine, and serotonin) (Fig. 1) [16–19]. The majority of studies on liver growth focus on clinically measurable endpoints, such as liver volume or weight, and the increase in these measures seen after PH or PVE is generally referred to as liver hypertrophy. However, preclinical studies on rodents have shown that the overall growth of the liver following PH or PVE depends not only on hepatocyte hypertrophy but also on hepatocyte hyperplasia [18, 20–22]. It has been shown in rats that the hepatocytes in the FLR proliferate in both PH and PVE, and the proliferating cell nuclear antigen index peaks in the FLR after PH and PVE at 24 and 48 h, respectively [23, 24]. After which, biliary epithelial cells, Kupffer cells, stellate cells, and sinusoidal endothelial cells also proliferate to restore the typical lobular architecture [25].

### Technique

**Fig. 2:**
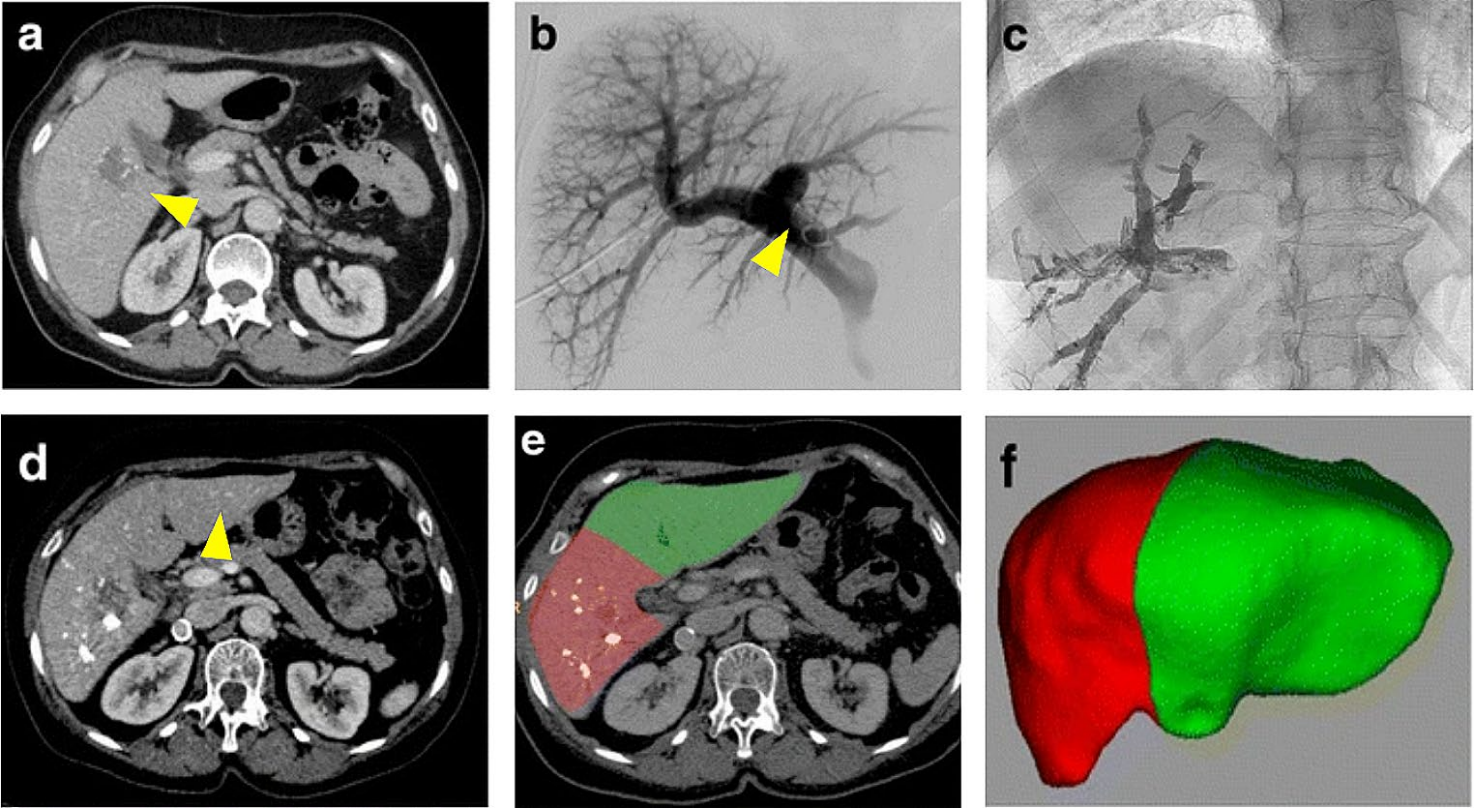
Imaging before and after PVE. **(a)** CT with contrast before PVE in a 67-year-old woman with metastatic colorectal cancer (arrowhead, right liver lobe). **(b)** Direct portography showing normal portal vein anatomy (arrowhead, portal vein). **(c)** Glue cast in the right portal vein branches demonstrating adequate embolization using an N-butyl-cyanoacrylate-lipiodol mixture. **(d)** CT imaging 30 days after right PVE showing growth of the left liver (arrowhead, left liver lobe). **(e, f)** CT volumetry after PVE illustrates an increase in FLR (red, right liver lobe; green, left liver lobe). Abbreviations: CT, computed tomography; FLR, future liver remnant; PVE, portal vein embolization. Images have been adapted from Luz et al., 2017 [30]. The figure has been created using Biorender.com

Prior to PVE, the FLR must be calculated to determine whether PVE is a necessary and appropriate treatment. A number of methods for calculating FLR may be used, including both volumetric and functional tests. A method to estimate the total required liver volume as a function of the patient’s size is provided by the following validated calculation based on a linear correlation between total liver volume (TLV) and body surface area (BSA): TLV = − 794.41 + 1267.28 × BSA (m^2^) [26]. Axial images from computed tomography and magnetic resonance imaging are the primary methods for volumetric assessment of the liver, both before and after resection (Fig. 2).

There are several techniques for accessing the portal vein for PVE. The less invasive methods include percutaneous approaches, where transhepatic puncture of the portal vein is executed using ultrasound and fluoroscopic guidance. The cutaneous approach is generally performed via an ipsilateral (i.e., same side as the PVE) or contralateral approach (i.e., opposite side from the PVE) [27]. An older surgical technique, often referred to as the transileocolic approach, may also be used, whereby direct puncture of the ileocolic venous branch is conducted through a lower right quadrant incision. No literature suggests that any of these techniques are contraindicated in the case of concurrent stem cell administration.

There are several embolic agents available for use in PVE. These include gelatin sponges, N-butyl-cyanoacrylate (NBCA), nitinol vascular plug, occlusion coils, polyvinyl alcohol, sodium tetradecyl sulfate, and trisacryl microspheres [28]. The two most commonly used agents are trisacryl microspheres and coils as well as a mixture of NBCA and lipiodol. Most agents are considered safe and have demonstrated low complication rates. Considerations taken into account when choosing an agent should include its safety profile, cost, ease of use, efficacy, and required fluoroscopy time [29].

## Clinical Challenges in Preoperative PVE

Increased resectability and decreased post-hepatectomy liver failure rates after PVE have been observed in appropriately selected patients. However, PVE is still limited by inadequate and protracted growth, especially in patients with specific risk factors. Several risk factors have been reported in the literature, such as a history of chemotherapy, underlying liver dysfunction, advanced age, and sarcopenia [31]. It is postulated that these factors contribute to a diminished functional capacity per unit volume of the liver. However, a recent meta-analysis by Soykan et al. revealed that there was no significant difference in the extent of hypertrophy following PVE between patients who did not undergo pre-procedural systemic therapy and those who received neo-adjuvant chemotherapy [31]. The pooled data, though, showed a high degree of heterogeneity. Patients with background liver fibrosis have also been reported to exhibit delayed or insufficient FLR growth after PVE [32]. Paradoxically, however, some studies have reported that background cirrhosis, which is the late stage of progressive hepatic fibrosis, does not significantly affect the extent of FLR growth [33, 34]. Importantly, regardless of the degree of FLR hypertrophy following PVE, patients with evidence of liver injury require a larger quantity of FLR following right or extended right hepatectomy, underscoring the need for improved hypertrophy techniques in this patient population [35].

Similarly, conflicting findings have been reported on the relationship between advanced age and FLR growth, with the majority of research revealing no correlation [31]. Overall, liver function seems to be uniquely protected in aging humans. While many other organs exhibit a significant age-related decline in structure and function, the liver exhibits relatively modest changes related directly to aging [36]. A 2015 case-control study found that patients 70 years and older did not have impaired liver growth after portal vein occlusion compared to patients under 70 years of age [37]. In the same study, however, age was associated with greater postoperative liver failure, which underscores the importance of liver quality and function and suggests that the magnitude of FLR growth in older patients needed to prevent postoperative failure may be greater compared to that in younger patients. Sarcopenia is a condition of low muscularity and low muscular strength that occurs most often in old age [38]. As opposed to advanced age, it has been consistently linked to inadequate FLR growth [39–42]. The relationship between sarcopenia and poor FLR growth following PVE is correlative [39–42], and further studies are needed to uncover the mechanisms underlying this association.

To date, advancements to PVE to further maximize liver growth have been largely mechanical in nature (i.e., right PVE extended to segment 4 branches [43], ALPPS [44, 45], and LVD [6]). The advantage of PVESA over these other techniques is that it may specifically address the critical need to improve FLR growth in a large number of patients with underlying liver fibrosis and comorbidities. Nonetheless, further investigations on the mechanisms underlying the relationship between the aforementioned risk factors and suboptimal liver growth following PVE are necessary to help inform clinicians in making decisions regarding which patients may most benefit from an enhanced PVE procedure, such as PVESA.

## Role of Stem Cells in Augmenting PVE

Although hepatocyte repopulation following resection of a healthy liver is mainly conducted by fully differentiated hepatocytes, which have a sufficient regenerative capacity to reconstruct the full volume of the liver, stem cells may play a bigger role in the context of massive acute liver injury or chronic liver disease [17, 21]. Typical regeneration in healthy livers involves hepatocyte hypertrophy and hyperplasia [18, 20–22]. Hepatocyte hypertrophy may sufficiently compensate for the loss of function because of small resections, but hepatocyte hyperplasia is necessary for resections involving more than 2/3 of the total liver volume. In the context of massive acute liver injury (≥ 80% loss of total liver volume) or chronic liver disease, the regenerative reserve of parenchymal hepatocytes becomes exhausted to the point that hepatic progenitor cells (HPCs)—the primary resident stem cells of the liver found in the canals of Hering—are activated [17, 21]. HPCs have the capacity to differentiate into hepatocytes and biliary epithelial cells, but their role in the reconstruction of hepatic architecture is not well established [21, 46]. Hence, the use of other stem cell types, which have the potential to not only differentiate into hepatocytes but also enhance hepatocyte proliferation through pro-proliferative signaling, has been explored in the last decades. Since up to 30% of patients undergoing PVE do not ultimately qualify for major resection because of malignant progression and/or inadequate liver growth, the use of stem cells to augment liver growth in a potentially shorter period could benefit a significant proportion of patients with liver malignancies.

### Stem Cell Types and Potential Mechanisms of Augmented Liver Growth

Stem cells can augment FLR growth achieved by PVE through their paracrine effects and by increasing the pool of cells that can differentiate into hepatocytes [47–51]. Stem cell types that have been used to augment FLR growth following PVE or PVL in animal studies include adipose tissue-derived mesenchymal stem/stromal cells (AMSCs), bone marrow-derived mesenchymal stem/stromal cells (BMSCs), unrestricted somatic stem cells (USSCs), and small hepatocyte-like progenitor cells (SHPCs). On the other hand, clinical studies of PVESA have utilized CD133 + BMSCs and hematopoietic stem cells (HSCs).

MSCs have immunomodulatory and multi-lineage differentiation capacities, making them an ideal candidate for various clinical applications. These cells are characterized by the expression of markers such as CD105, CD73, and CD90 [52]. MSCs can be isolated from a wide variety of tissue types, expanded in vitro without significant changes in their properties, and delivered via autologous or allogeneic transplantation because of their low immunogenicity [53]. In the context of PVESA, preclinical studies have utilized AMSCs and BMSCs, while clinical studies have only utilized autologous BMSCs. The ability of AMSCs and BMSCs to differentiate into hepatocyte-like cells has been demonstrated in several preclinical in vivo studies [54, 55]. Umbilical cord-derived MSCs (UMSCs) have also been implanted in rats with fibrotic livers and have been shown to differentiate into cells that express hepatocyte-specific markers, such as albumin, α-fetoprotein, and cytokeratin 18 [56, 57]. With the support of specific growth factors, such as EGF, fibroblast growth factor, HGF, oncostatin M, and trichostatin A, MSCs from different tissues can also be induced in vitro to differentiate into cells with liver-specific morphology and function [58–60]. However, in some studies, only a small proportion of transplanted MSCs differentiate into hepatocyte-like cells, suggesting that they primarily promote liver regeneration through mechanisms other than direct differentiation into hepatocytes [61, 62]. Indeed, MSCs can also enhance the repair of injured liver tissue by exerting paracrine effects, which involve the secretion of soluble factors and modulation of inflammation [63].

MSCs have been shown to stimulate hepatocyte proliferation and inhibit hepatocyte apoptosis in rodent models of acute liver injury [64–68]. Secretory products of various MSCs that have been implicated in hepatocyte proliferation include HGF, VEGF, interleukin-10 (IL-10), and PGE_2_ [66, 69, 70]. HGF is a growth factor that stimulates the proliferation of hepatocytes and several other cell types by activating the c-Met receptor [71], and it has been shown that UMSCs can promote liver regeneration in rats with acute-on-chronic liver failure through an increase in HGF expression [69]. VEGF is a growth factor that promotes hepatocyte proliferation by stimulating the regeneration of endothelial cells, and consequently, the liver sinusoids [72, 73]. It has been shown to support hepatocyte proliferation in rodent models of acute liver injury [67] and PH [72, 73]. IL-10 is an anti-inflammatory cytokine that has been implicated in mediating the immunomodulatory and regenerative effects of AMSCs in the context of acute kidney injury [66], while PGE_2_ is a vasoactive factor that has been shown to mediate the anti-apoptotic and pro-proliferative effects of BMSCs towards hepatocytes in a mouse model of acute liver failure [70].

The capacity of MSCs to attenuate liver fibrosis and inflammation has also been reported in several studies [74, 75]. BMSCs can modulate the proliferation of hepatic stellate cells [74], which are responsible for the production of collagen-rich extracellular matrix (ECM) in the liver in response to injury, and regulate the expression of matrix metalloproteinases (MMPs) [75], which mediate the degradation and remodeling of ECM in fibrotic livers. MSCs also exert immunomodulatory activity that affects both the innate and adaptive arms of the immune system. In animal models of liver disease, BMSCs promoted the polarization of macrophages to the immunomodulatory M2 phenotype through the secretion of anti-inflammatory cytokines, such as chemokine ligand 1 and IL-10 [76, 77]. Various MSC types have also been shown to promote the generation of regulatory T-cells and suppress the activation of effector T-cells through the secretion of several molecules, such as inducible NO synthase, HGF, MMPs, PGE_2_, and transforming growth factor beta (TGF-β) [53].

HSCs represent a small population of self-renewing, multipotent cells that lie at the apex of the hematopoietic system. These cells are characterized by the expression of CD34 and CD133, and they have been used in several clinical studies of liver regeneration because of their plasticity [78]. HSCs are hypothesized to aid in liver regeneration primarily through hepatocyte differentiation and hepatocyte fusion [79]. HSCs are isolated from the patient prior to PVE via leukapheresis of the peripheral blood or directly from bone marrow. Given that many HSC investigations employ granulocyte colony–stimulating factor for mobilization into the peripheral blood, it is important to understand how the administration of this growth factor affects liver regeneration. Granulocyte colony–stimulating factor infusion in patients is often accompanied by an increase in the serum levels of growth factors that play a role in liver regeneration, such as HGF and VEGF [80]. Hence, this may be a confounding factor in understanding the mechanism of PVESA involving HSCs.

USSCs, also known as MSC progenitors, are stem cells that can be isolated from cord blood and display a broad differentiation potential for ectodermal, mesodermal, and endodermal cell types. Similar to MSCs, these cells also have relatively low immunogenicity, which allows allogeneic transplantation [81]. USSCs have been shown to differentiate into hepatocytes [50], although it is unclear whether they can generate secretory products that can support hepatocyte growth.

SHPCs, also known as small hepatocytes, are a special subset of hepatic progenitor cells that emerge in rat livers treated with retrorsine and a 70% PH. Retrorsine is a pyrrolizidine alkaloid that binds DNA and inhibits hepatocyte proliferation. In livers treated with retrorsine, SHPCs can be distinguished from surrounding hepatocytes through their smaller and darker-staining nuclei, as well as fine fat droplets in the cytoplasm [82]. These cells represent a less differentiated population and have been reported to be more resistant to several forms of toxic injury [83]. Similar to HSCs, these stem cells are hypothesized to aid in liver regeneration primarily through hepatocyte differentiation.

### Preclinical in Vivo Studies of PVESA

The efficacy of PVESA has been explored in several preclinical in vivo studies using murine, swine, and ovine models, as shown in Table 1.

The first animal studies focused on the effect of PVESA in the context of regenerating healthy livers. Liska et al. first evaluated the intraportal injection of allogeneic BMSCs stained with 5-bromo-2-deoxyuridine following PVE in 6-week-old piglets and found that the BMSC group demonstrated a significantly larger non-ligated lobe than did the control group on the third and seventh days after surgery. Although the BMSC group had 30% larger non-ligated lobes on average at the end of the experiment, the difference between the groups lost statistical significance on the 10th and 14th days, suggesting that the added benefit of BMSC augmentation to liver growth is more pronounced early during hepatic regeneration. Both groups followed similar patterns of serum aspartate transaminase, alanine transaminase, alkaline phosphatase, bilirubin, and gamma-glutamyltransferase. At the end of the experiment, the Ki-67 proliferative index was practically the same between the two groups and equal to levels found in a normal liver. Few 5-bromo-2-deoxyuridine-stained cells were found in the liver, which is why the authors concluded that the injected BMSCs enhanced liver regeneration mainly through the establishment of a supportive micromilieu for pre-existing liver hepatocytes instead of repopulating the liver through differentiation into hepatocytes [47].

In another study using a swine model, the intraarterial injection of human adipose tissue–derived stromal vascular fraction—a heterogeneous mix of various cell populations, including AMSCs, endothelial progenitor cells, and vascular smooth muscle cells—normalized the transcriptomic profile of the regenerating liver after 9 days, while the control group had 39 differentially expressed genes related to fibrosis and inflammation. However, the intravenous administration of hydrocortisone in the adipose tissue–derived stromal vascular fraction group to prevent immune rejection could be a confounder in the observed reduction in fibrosis and inflammation. The research is also constrained by a small sample size, and the authors suggested increasing the number of biological replicates and including additional time points for succeeding studies [84].

Ghodsizad et al. reported that human USSCs can support hepatic regeneration in an ovine model by differentiating into hepatocytes within 4 weeks. However, liver tests and volumetry were not reported, which limits the assessment of the treatment’s impact on hepatic regeneration [50].

**Fig. 3:**
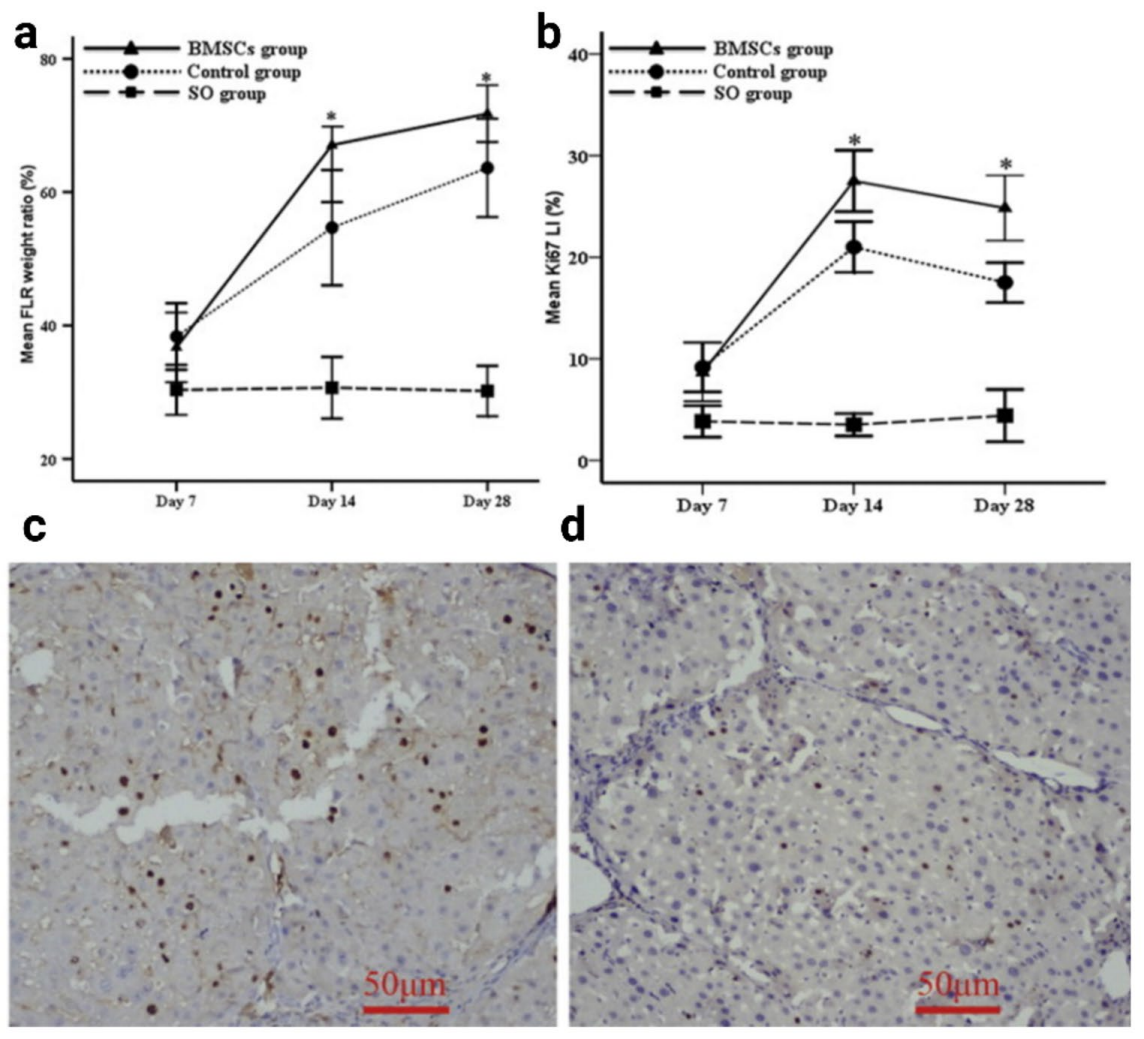
Liver growth after PVESA in cirrhotic rats. **(a)** Ratios of FLR versus total liver weight as well as **(b)** Ki-67-positive cells among the three groups were significantly different (*p* < 0.001), with the BMSC-treated group showing the highest values at days 14 and 28 after surgery (*, *p* < 0.001; BMSCs, bone marrow-derived mesenchymal stem cells; FLR, future liver remnant; LI, labeling index; SO, sham operation). **(c/d)** Representative immunohistochemistry slides for the BMSC-treated (**c**) and PVE-only control (**d**) groups show higher expression of Ki-67 (brown nuclear stains) in the BMSC-treated liver. Images have been adapted from Li et al., 2013 [48]. The figure has been created using Biorender.com

Given that many liver cancer patients have varying degrees of chronic liver damage, it is imperative to evaluate PVESA in the context of chronic liver disease. Li et al. investigated the intraportal injection of autologous PKH26-labeled BMSCs in cirrhotic Sprague-Dawley rats and found that the BMSC group had a significantly higher FLR to total liver weight ratio (Fig. 3), Ki-67 proliferation index, and serum albumin level than did the control group at 14 and 28 days after PVE. The BMSC group also had a decreased level of total bilirubin as well as hydroxyproline and collagen content in the liver. Gene expression studies revealed the FLR of the BMSC-treated group had a significantly higher expression of *VEGF*, *HGF*, *IL-10*, and *MMP-9*. The PKH26-labeled BMSCs differentiated into functional hepatocytes and expressed hepatocyte-specific markers, such as α-fetoprotein, cytokeratin 18, and albumin. Overall, this study supports the dual role of MSCs in augmenting liver growth after PVE, as it has been shown that they differentiate into hepatocytes and improve the local microenvironment by reducing fibrosis in a liver fibrosis model [48]. Nonetheless, it is important to note that on days 14 and 28, the BMSC group had significantly higher aspartate transaminase and alanine transaminase than the control group. Although cell emboli were not detected in this study, the observed elevation in liver enzymes may be attributed to the presence of these masses, since they can cause significant ischemia and elevate serum aminotransferases once they reach the hepatic sinusoids [85].

Lastly, Miyazaki et al. evaluated the splenic implantation of AMSCs and SHPCs after liver irradiation and PVL and found that both cell types had successfully migrated and engrafted into the irradiated liver after 8 weeks [51]. Notably, SHPCs showed more significant hepatic proliferation, but AMSCs did not, suggesting that the activity of MSCs may differ in the context of the regeneration of an irradiated liver.

**Table 1:** Preclinical in vivo studies on PVE/PVL and stem cell augmentation

Stem cell	Model	Intervention	Results	Reference
AMSC and SHPC, allogeneic	Fischer 344 rats	Splenic implantation after liver irradiation and PVL	After 8 weeks, both AMSCs and SHPCs had successfully migrated and engrafted into the irradiated liver. SHPCs showed significant hepatic proliferation.	Miyazaki et al., 2013 [51]
ASVF, human	Polish white pigs	Intraarterial injection after PVL	After 9 days, the control pigs had differentially expressed genes related to liver fibrosis and inflammation. BMSC patients had no differentially expressed genes, indicating a properly regenerated liver remnant.	Bartas et al., 2018 [84]
BMSC, allogeneic	Piglets	Intraportal injection after PVE	BMSC-treated animals demonstrated significantly higher liver growth at 3 and 7 days after operation. At 14 days, the BMSC group had 30% larger non-ligated lobes compared to the control, albeit not statistically significant.	Liska et al., 2009 [47]
BMSC, autologous	Cirrhotic Sprague-Dawley rats	Intraportal injection after PVE	FLR to total liver weight ratio was significantly higher at 14 and 28 days after operation for the BMSC group. Albumin was significantly increased in the BMSC group from the 7th day onward, while total bilirubin and aspartate aminotransferase were significantly decreased from the 14th day onward.	Li et al., 2013 [48]
USSC, human	Sheep	Intraportal injection after PVE	After 4 weeks, the transplanted cells differentiated into hepatocytes and produced albumin.	Ghodsizad et al., 2012 [50]

*Abbreviations* AMSC, adipose tissue–derived mesenchymal stem cell; ASVF, adipose tissue-derived stromal vascular fraction; BMSC, bone marrow–derived mesenchymal stem cell; FLR, future liver remnant; PVE, portal vein embolization; PVL, portal vein ligation; SHPC, small hepatocyte-like progenitor cell; TLW, total liver weight; USSC, unrestricted somatic stem cell

### Clinical Studies on PVESA

The efficacy of PVESA has been tested in several clinical studies in patients with liver tumors, such as CCA, HCC, mCRC, and metastatic neuroendocrine tumors, as shown in Table 2.

The benefit of PVESA in patients with liver malignancy was first reported by am Esch et al. in a small study involving three cases treated with intraportal injection of autologous CD133 + BMSCs after PVE and three controls who underwent PVE alone. The BMSC-treated group had a 2.5-fold higher mean daily gain in FLR volume compared to the control group (*p* < 0.01), and all BMSC-treated patients had sufficient FLR for liver resection. Two control patients did not undergo resection due to disease progression in the FLR [86].

Furst et al. expanded on the first study by increasing the number of patients to 6 BMSC-treated cases and 7 PVE-only controls. Similarly, the BMSC-treated group had a 2.3-fold higher mean daily gain in FLR volume compared to the control group (*p* = 0.03). All BMSC-treated patients had sufficient FLR and underwent extended hepatectomy. Six PVE-only controls underwent extended hemihepatectomy, but their time to surgery was 1.7x longer for the control group versus the treated group (*p* = 0.057), which supports the hypothesis that PVESA can accelerate the growth of the FLR. One treated and one control patient had minimal dislocation of the embolic agent [87].

To evaluate the functional and survival outcomes after extended right hepatectomy, am Esch et al. conducted a retrospective study involving 11 BMSC-treated cases, 11 PVE-only cases, and 18 controls with no presurgical FLR expansion. They found no significant differences among the groups in terms of functional parameters on postoperative day 7, but FLR expansion was found to be positively associated with overall survival. A post hoc analysis revealed that the BMSC group (*p* = 0.028) had significantly longer survival compared with controls. The median overall survival durations were 27, 20, and 6 months in the PVESA, PVE, and control groups, respectively. There were no complications associated with PVE, alone or with PVESA, before the extended right hepatectomy. Two PVESA and three PVE-alone patients were not able to undergo extended right hepatectomy because of unresectable disease or physical impairment [88].

Aside from CD133 + BMSCs, HSCs have also been utilized in clinical investigations of PVESA. Treska et al. reported the benefit of autologous CD133 + HSCs after PVE in 5 cases of HCC or mCRC. Increases in FLR volume (> 30%) occurred in all cases 2–4 weeks following PVESA. Three patients had sufficient FLR and underwent resection. One patient had severe intra-abdominal adhesions, while the last patient experienced tumor progression, which hampered resection [49]. Similarly, Canepa et al. reported that the intraportal injection of autologous CD133 + HSCs after PVE results in a higher FLR volume gain compared to that in PVE-only patients [89].

Lastly, Han et al. evaluated the intraportal injection of HSCs after PVE in a randomized trial and found that the daily hepatic volume growth in the HSC-treated group was significantly higher than that in the PVE-only group [90].

**Fig. 4:**
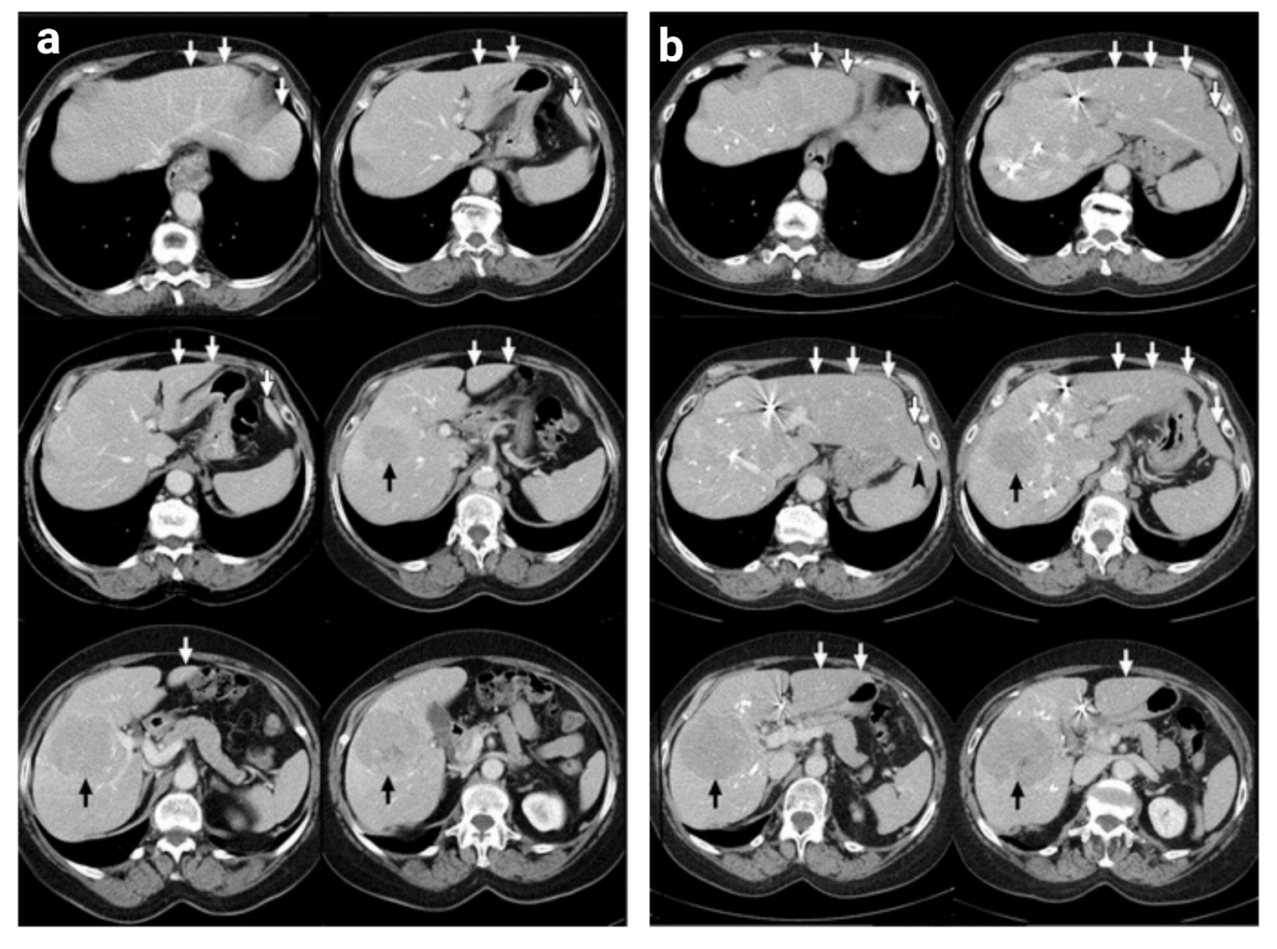
Liver growth after PVESA in a patient with HCC. **(a)** Axial helical computed tomography scans before and **(b)** 14 days after PVE and intraportal autologous CD133 + BMSC application revealed marked growth of segments II and III, marked by white arrows (black arrowhead, dislocated cyanoacrylate-to-iodinized oil particle; black arrow, hepatocellular carcinoma). Images have been adapted with permission from Fürst et al. 2007 [87]. The figure has been created using Biorender.com

Overall, clinical studies demonstrate that stem cell augmentation is a generally safe procedure that enhances liver growth after PVE. However, larger trials are needed to validate the clinical benefit seen in the aforementioned studies. Moreover, none of the clinical investigations have evaluated the behavior of the delivered stem cells and the expression of relevant signaling pathways in the FLRs. Hence, more extensive biological studies must be conducted in further clinical studies to better shape our understanding of the underlying mechanisms of liver regeneration in the context of PVESA, especially in humans(Fig. 4).

**Table 2:** Clinical studies on PVESA

Stem cell	Intervention	Tumor	Study design	No. of patients	Results	Reference
Autologous BMSC, CD133+	Intraportal injection of 2.4–12.3 × 10^6 cells/patient after PVE	CCA, HCC, mCRC, mNET	Prospective study	3 BMSC-treated cases, 3 PVE controls	Treated group had a 2.5-fold higher mean daily gain in FLR volume compared to the control group (9.87 ± 1.72 mL versus 4.03 ± 0.47 mL, *p* < 0.01).	am Esch et al., 2005 [86]
Autologous BMSC, CD133+	Intraportal injection of 2.4–12.3 × 10^6 cells/patient after PVE	CCA, HCC, mCRC, mNET	Prospective study	6 BMSC-treated cases, 7 PVE control	Treated group had a 2.3-fold higher mean daily gain in FLR volume compared to the control group (9.5 ± 4.3 mL versus 4.1 ± 1.9 mL, *p* = 0.03). Time to surgery was 1.7x longer in the control group than in the treated group (45 ± 21 versus 27 ± 11 days, *p* = 0.057).	Fürst et al., 2007 [87]
Autologous BMSC, CD133+	Intraportal injection after PVE	CCA, HCC, mCRC, mNET	Retrospective study	11 BMSC-treated cases, 11 PVE-only cases, 18 controls	FLR expansion was positively associated with overall survival. The BMSC group (*p* = 0.028) had significantly longer survival compared with controls.	am Esch et al., 2012 [88]
Autologous, HSC, CD133+	Intraportal injection of 1.2–12.2 × 10^7 cells/patient after PVE	HCC, mCRC	Case series	5 HSC-treated cases	Sufficient increase in FLR volume (> 30%) occurred in all five patients between 2–4 weeks following PVE with HSC augmentation.	Treska et al., 2013 [49]
Autologous, HSC, CD133+	Intraportal injection after PVE	Not specified	Prospective study	6 BMSC-treated cases, 10 PVE controls	HSC-treated patients showed a higher FLR volume gain compared to PVE-only patients (*p* < 0.001).	Canepa et al., 2013 [89]
Autologous, HSC	Intraportal injection after PVE	HCC	Randomized trial	10 HSC-treated cases, 10 PVE controls	Daily hepatic volume growth in the HSC-treated group was significantly higher than in the PVE-only group (4.9 versus 1.1, *p* < 0.05).	Han et al., 2014 [90]

*Abbreviations* BMSC, bone marrow-derived mesenchymal stem cell; CCA, cholangiocarcinoma; CD, cluster of differentiation; FLR, future liver remnant; HCC, hepatocellular carcinoma; HSC, hematopoietic stem cell; mCRC, metastatic colorectal cancer; mNET, metastatic neuroendocrine tumor; PVE, portal vein embolization

## Future Directions

PVESA has been shown to enhance the degree and rapidity of FLR growth in various studies, but this technique has not yet seen widespread adoption. To facilitate the clinical translation of PVESA, several issues must be addressed. These include the identification of optimal stem cell types, refinement of stem cell collection and administration, appraisal of novel stem cell technologies for potential integration, and evaluation of underlying mechanisms to maximize FLR growth and minimize tumor progression.

### Stem Cell Selection

The stem cell types utilized in clinical studies of PVESA have been limited to BMSCs and HSCs. However, there is still no definitive standard regarding which type of stem cells should be utilized for liver regeneration in the context of PVE. BMSCs are highly suitable for autologous transplantation, but the isolation method is invasive and may cause adverse effects, such as injury and site inflammation. Moreover, it should be noted that although the research surrounding adult stem cells for FLR growth is promising, adult stem cells may not be versatile and durable, especially in older patients. Alternatively, UMSCs, which may be obtained from tissue that might otherwise have been disposed, have been shown to have a lower immunogenicity and higher self-renewal and differentiation capacity than BMSCs. In addition, because these cells originated from early-phase tissue, they do not experience an age-related decline in proliferation that weakens the therapeutic effect of MSCs. AMSCs are also easier to obtain than BMSCs, but they have been shown to have lower proliferative and immunomodulatory capacity [53]. On the other hand, HSCs may also be mobilized from the bone marrow to the peripheral blood to facilitate a less invasive isolation process. However, mobilization typically involves the administration of granulocyte colony–stimulating factor, which may be a confounding factor in stimulating liver growth. Comparative studies are necessary to evaluate the performance of various stem cell types.

### Stem Cell Collection and Administration

Although all clinical studies on PVESA have used autologous stem cell transplantation, allogeneic transplantation is emerging as an attractive alternative because of the low immunogenicity of several stem cell types. In many patients with co-morbid conditions, the type and amount of stem cells may be compromised, and the time it could take to obtain a sufficient number of stem cells could cause physicians to miss the window of treatment for patients with liver malignancies and chronic liver disease [53]. Although most research studies have administered stem cells into the main portal vein following PVE, the optimal dosage and route of stem cell administration remain unknown. At present, it is unclear whether systemic administration is sufficient. The timing of administration, potentially at multiple time points, may improve stem cell engraftment into the liver since a major limitation of PVESA is the rapid washout of the stem cells from the delivery site. In the case of MSCs, most studies have shown that less than 5% of administered cells are present in the target tissue a few hours after transplantation [91]. Hence, different techniques for stem cell collection and administration should be investigated thoroughly to determine which approach produces the greatest benefit with the least risk to the patient.

### Novel Stem Cell Technologies

Novel stem cell technologies are currently being developed to improve the efficacy of stem cells for liver regeneration. Advancements in this area are mostly focused on MSCs and include techniques such as priming, genetic manipulation, and extracellular vesicle (EV)-based therapy [92, 93]. Priming is a pre-conditioning process that involves the exposure of cells to cytokines, growth factors, and selected microenvironments to enhance their survival and augment their valuable properties. In the case of MSCs, studies have demonstrated that various priming strategies can promote their survival and amplify their immunomodulatory secretions. These strategies include the use of cytokines, growth factors, hypoxia, and three-dimensional culture conditions [94]. In the context of liver regeneration, it has been shown that the exposure of AMSCs to low-dose lipopolysaccharide (LPS)—a potent endotoxin that induces the release of proinflammatory cytokines—for 24 h can increase their expression of *IL-6*, *TNF-α*, *HGF*, and *VEGF*, and the infusion of LPS-conditioned media can significantly enhance liver regeneration following PH in mice [95]. Priming of BMSCs with TGF-β—a major fibrogenic cytokine—has been shown to enhance their ability to engraft into the liver and to reduce inflammation and necrosis in mice with liver injury [96]. Similarly, priming of BMSCs with melatonin has been shown to enhance their ability to home and to reduce lipid accumulation, fibrosis, and hepatocyte apoptosis in rats with induced liver fibrosis [97].

Another emerging strategy is the genetic modification of MSCs through the use of viral vectors. Studies have shown the overexpression of HGF in BMSCs enhanced the BMSCs’ ability to prevent liver failure and reduce mortality in rats with small-for-size liver grafts [98] as well as enhance their ability to attenuate liver injury in rats with induced liver fibrosis [99]. The overexpression of hepatic nuclear factor 4 alpha—a transcription factor that plays an important role in hepatocyte maturation—has been shown to enhance the ability of BMSCs to reduce inflammation and attenuate liver injury in mice with induced liver fibrosis [100]. In another study, overexpression of CC motif chemokine receptor 2—the receptor for monocyte chemoattractant protein-1—enhanced the ability of UMSCs to localize to the liver, reduce inflammation, and promote recovery in mice with acute liver failure [101]. While the aforementioned studies did not report major adverse effects associated with the use of viral vectors, it is still important to remain vigilant regarding potential risks, such as immunogenicity and vector-related toxicity.

EVs refer to lipid membrane vesicles that contain bioactive cargoes (e.g., proteins, lipids, nucleic acids) and are released by cells into the extracellular space to mediate intercellular communication and elicit diverse biological responses in recipient cells [102]. Exosomes refer to a subset of vesicles that are smaller than 200 nm. Although numerous cell types have been documented to secrete therapeutically active EVs, MSC-derived EVs are the most advanced in preclinical and clinical studies due to the well-established therapeutic benefits of MSC [102]. EVs derived from different MSC types have been shown to promote liver regeneration in preclinical models of liver injury via modulation of inflammation and attenuation of oxidative stress [103–105]. UMSC-derived EVs have been shown to modulate the expression of CD154—a member of the TNF superfamily and a stimulant of immune response—in intrahepatic CD4 + T-cells in mice with liver ischemia/reperfusion injury [103]. BMSCs have also been shown to regulate the miRNA content of the exosomes within the hepatic microenvironment and down-regulate the expression of CXC motif chemokine ligand 8—a pro-inflammatory cytokine elevated in patients with acute liver failure [104]. Glutathione peroxidase 1-containing exosomes from UMSCs have also rescued mice from induced liver failure by reducing oxidative stress and hepatocyte apoptosis [105]. Similarly, EVs have also been shown to promote liver regeneration following PH. Exosomes derived from UMSCs contain miRNAs that can promote liver regeneration after PH in rats [106]. EVs are an attractive alternative for MSCs due to their simpler storage and administration compared to MSCs. Nevertheless, further research is needed to resolve issues surrounding EVs, such as biodistribution, pharmacodynamics, and cellular fate post-uptake.

### Mechanistic Studies and Larger Trials

Despite the potential of PVESA, obstacles such as limited understanding of its mechanisms and the lack of standardized protocols hinder its adoption in interventional radiology. The exact mechanisms by which each stem cell type enhances FLR growth following PVE are not completely understood. Moreover, although current studies do not show an increased risk of cancer progression with PVESA, more investigations are needed to thoroughly understand this process. Most animal studies on PVESA have used models without liver tumors, which prevents an expansive evaluation of the effects of PVESA on tumor biology. The establishment of orthotopic models for PVESA could provide baseline information and mechanistic insights that can address the concerns surrounding PVESA’s safety and efficacy. Additionally, further insight into the specific factors that could support stem cell–induced FLR growth and inhibit tumor progression could give rise to novel approaches, such as the use of biologics and combination therapies. Nine years have elapsed since the last publication of the results of a randomized trial on PVESA. A clinical investigation of the efficacy of autologous CD133 + HSCs in augmenting liver growth after PVE patients with mCRC was recently completed in 2021 (NCT03803241); the results are still pending. Ultimately, randomized-controlled clinical trials with greater numbers of participants and more in-depth biological studies of human tissue samples must eventually be performed to validate the safety and efficacy of PVESA, especially within the context of alternative FLR techniques, such as LVD and ALPPS.

## Conclusion

PVESA is an exciting method for enhancing FLR growth before resection. Any technique that enhances the magnitude and rate of FLR hypertrophy will necessarily translate into an increased number of patients with liver cancer who may undergo curative resection while minimizing the risk of post-operative liver insufficiency and death. Much remains unknown regarding the mechanism by which stem cells enhance growth following PVE, yet this procedure has been shown repeatedly to increase FLR size compared to traditional PVE. The utility of PVESA within the context of newer techniques used to induce greater degrees of FLR hypertrophy (i.e., LVD and ALPPS) needs to be elucidated. An advantage of MSCs is their ability to attenuate liver inflammation and fibrosis. Many patients with liver malignancy will have underlying chronic liver disease. While there are multiple techniques capable of inducing FLR hypertrophy prior to major liver surgery, PVESA may also address the underlying quality of the FLR. Thus, further elucidation of the driving mechanisms of PVESA may unlock even more advanced and practical treatments to enhance FLR volume and perhaps address underlying liver disease.

## Data Availability

Not applicable.

## Abbreviations

AMSC: Adipose tissue-derived mesenchymal stem/stromal cell
ASVF: Adipose tissue–derived stromal vascular fraction
BMSC: Bone marrow–derived mesenchymal stem/stromal cell
CCA: Cholangiocarcinoma
ECM: Extracellular matrix
EGF: Epidermal growth factor
EV: Extracellular vesicle
FLR: Future liver remnant
HCC: Hepatocellular carcinoma
HGF: Hepatocyte growth factor
HPC: Hepatic progenitor cells
HSC: Hematopoietic stem cell
IL: Interleukin
LPS: Lipopolysaccharide
mCRC: Metastatic colorectal cancer
MMP: Matrix metalloproteinase
mNET: Metastatic neuroendocrine tumor
MSC: Mesenchymal stem/stromal cell
NBCA: N-butyl-cyanoacrylate
NO: Nitric oxide
PGE_2_: Prostaglandin E_2_
PH: Partial hepatectomy
PVE: Portal vein embolization
PVESA: PVE with stem cell augmentation
SHPC: Small hepatocyte-like progenitor cell
TGF: Transforming growth factor
TLV: Total liver volume
UMSC: Umbilical cord-derived mesenchymal stem/stromal cell
USSC: Unrestricted somatic stem cell
VEGF: Vascular endothelial growth factor
